# Design, implementation, and management of an international medical device registry

**DOI:** 10.1186/s13063-021-05821-5

**Published:** 2021-11-25

**Authors:** Herbert Mauch, Jasmin Kaur, Colin Irwin, Josie Wyss

**Affiliations:** 1 Cochlear AG, Basel, Switzerland; 2 Cochlear France SAS, Toulouse, France; 3 Cochlear Ltd., Sydney, Australia

**Keywords:** International registry, Medical device, Cochlear implant, Quality of life, SSQ, IROS

## Abstract

**Background:**

Registries are powerful clinical investigational tools. Although in hospitals registries may be mandated, industry-sponsored, international registries are voluntary and therefore can require clearer objectives and more planning. The registry also needs sufficient resources and appropriate measurement tools to motivate long-term participation and ensure success.

**Methods:**

We summarize our learnings from 10 years of running a medical device registry that surveys patient-reported benefits of hearing implants.

**Results:**

We enlisted 77 participating clinics globally, who actively recruited a total of more than 1500 hearing implant users. We identified the stages in developing a registry specific to hearing loss. Furthermore, we report the challenges and successes in design and implementation and make recommendations for future registries.

**Conclusions:**

Data collection infrastructure needs to be kept up to date throughout the defined registry lifetime, and it is essential to oversee data quality and completeness. Compliance at registry sites is important for data quality and needs to be weighed against the cost of site monitoring. To motivate sites to enter data accurately and expeditiously, we facilitated easy access to their own data which helped to support their clinical routine.

**Trial registration:**

ClinicalTrials.gov NCT02004353. 9^th^ December 2013.

## Introduction

A registry has been defined as ‘an organized system that uses observational methods to collect defined clinical data under normal conditions of use relating to one or more medical devices to evaluate specified outcomes for a population defined by a particular disease, condition, or exposure and that serves predetermined scientific, clinical or policy purpose(s)’ [20]. There are many types of medical registries, most of which are concerned with drug safety and efficacy, or are disease-specific registries, including rare diseases, or based on medical devices focused on safety and effectiveness. Most registries are supported as part of a regional or national mandate. Thorough overviews to developing a safety registry can be found in several publications [17, 19, 38]. Advice is also specifically available for starting a medical device registry [3, 24, 26, 27]. It is rare for a patient-related outcome registry to be both international- and corporate-sponsored, i.e. it is unusual for a corporation to take full responsibility for funding and logistics. In 2015, there were 1028 registries listed in the EU, of which only 13 were multinational. Eighty-three were for medical devices but only five of these, i.e. 3%, were sponsored by a corporation [38].

Cochlear Ltd. (Australia) manufactures several kinds of hearing implants, including bone conduction hearing solutions (‘Baha’), cochlear implants (CI), auditory brainstem implants (ABI) and middle-ear implants (note the latter are no longer available). These devices serve to overcome various dysfunctions in the auditory system, from the outer to the inner ear and even further up the auditory pathway. These solutions all restore sensitivity to sound. However, of course, the main aim of the treatment is restoring functional hearing, i.e. speech understanding [21]. Probing speech understanding capacity has been incorporated into generic health utility measures such as the Health Utility Index (HUI) [13]. This allows the benefit of a hearing implant to be calibrated against interventions for other health attributes. Disease-specific questionnaires for hearing loss, such as the Speech Spatial Qualities (SSQ) [14] have also been developed to allow more fine-tuned measures, for example, to allow comparison between devices.

We note that cochlear implantation is generally accepted to be the standard of care for profound neurosensory hearing loss where conventional acoustic hearing aids are not sufficient to restore functional speech understanding [7]. Similarly, other technologies such as Baha and middle-ear implants are used where conventional sound-amplifying acoustic hearing aids are not able to overcome physical barriers between the outer and inner ear.

It was a pioneering endeavour for, the company’s subsidiary, Cochlear AG (Switzerland) to consider establishing an international registry in 2009, as the only registry at the time was a national CI registry that had operated within Switzerland since the 1990s. The Swiss CI registry collected performance and safety data from clinics. However, it did not involve subjective patient-reported outcome measures [6, 33]. At its inception, the primary aim of Cochlear’s registry was to produce a real-world view of the impact of CI on hearing ability and QoL in daily life, a topic that continues to be of interest to researchers [1], by reporting on self-assessed benefits following hearing-implant treatment.

The formal name given to the Cochlear registry was the Implant Recipient Observational Study (IROS) [ClinicalTrials.gov NCT02004353]. The concept for the registry arose from the realization that the available clinical evidence from around the world supporting the benefits of CI did not lend itself to meta-analysis. For example, outcomes were not generalizable due to the limited inclusion criteria for small cohorts with relatively short observation times. Typically, data was diverse and thus difficult to collate and analyse collectively, especially hearing treatment outcome data, such as speech recognition measures or non-standardized patient-reported outcome measures used routinely across clinics and cultures. Prior to our registry, it was impossible to apply outcome data to broader populations, and any group data with diversified input could only be reported as results of strictly controlled clinical trials.

The registry would have the advantage over clinical trials involving cross-culturally adapted patient-reported outcome measures allowing broader participation by clinics, and their treatment groups, which are representative of less biased target populations [35, 38].

This would be best supported as an international registry using widely accepted questionnaires. The HUI instrument was already available in many languages, and the SSQ was also available in some language versions. One of the first tasks was to ensure further validated translations and cultural adaptations of the SSQ questionnaire in collaboration with the author developer [14] and for the HUI Mark III version in collaboration with HUI Inc. (Canada). These two questionnaires served as the primary endpoints for IROS. Both these metrics enable the capture of real-world experience and treatment benefits for implant users. To characterize the nature of hearing loss and further help the definition of user profiles versus the primary endpoints, we collected clinically relevant data points, such as aetiology, duration of hearing loss, progression of hearing loss and type and degree of hearing loss.

Unlike mandated registries with a primary focus on safety aspects often independently conducted, the Cochlear IROS directly fed into Cochlear’s postmarket vigilance system and its related activities. In this sense, the registry was complementary to these processes and information, and vice versa in regard to regulatory requirements to ensure unique reporting of events.

**Fig. 1:**
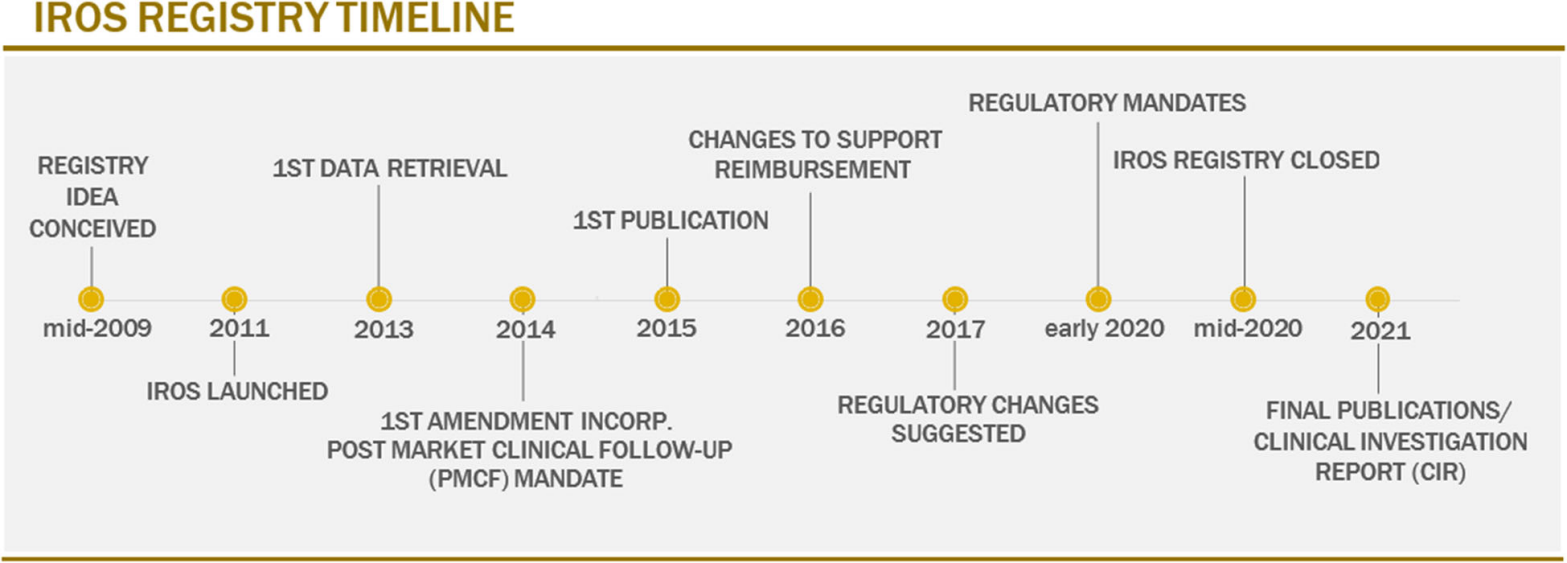
Flow diagram depicting IROS from conception to closure

Here, we report our experience and lay out a framework for crucial decisions that need to be taken in the pursuit of efficient data collection via a registry, ultimately leading to useful data analysis and reporting. The details we report are particularly relevant when conceptualizing the potential that they offer in creating better registries using modern data collection methods that reduce, or even eliminate, the workload on participating sites. Indeed, this burden on busy centres was identified as one of the major challenges faced during the operation of IROS, the first large hearing implant device registry using electronic data capture. We comprehensively present details relating to decision-making in the design, implementation, management, follow-up and the ongoing decision to continue or close a registry. Note that the IROS registry was closed mid-2020, see Fig. 1 for the timeline. The registry plan together with the results has been reported on ClinicalTrials.gov [NCT02004353]. Peer-reviewed publications reporting on outcomes from the data collection efforts during the conduct of IROS are listed under the ‘Outcomes’ section.

## Design

### Purpose and primary aims

The type of evidence defines the purpose of a registry, whether it be to collect real-world evidence or yield safety data for regulatory certifying bodies, federal and local funding authorities and other healthcare agencies, or to provide support for clinical or market access activities. Each will have its own set of advantages and limitations [35].

Regardless of the type of registry, there will always be shifting sub-goals as stakeholders may request broader application of the registry, such as mining of available data for specific clinical or product development purposes. It is, therefore, important to maintain a clear focus as to the fundamental aim of the registry.

As the IROS collected data over several years, we were able to survey hearing outcomes for recipients of new implantable hearing devices as they became available. Furthermore, we realized that data could equally be collected for adolescents (> 10 years of age) who were able to complete the patient-reported outcome measure questionnaires. Collecting similar data for children < 10 years of age is more complex and involves surveying parental perspectives and observations. Under a parallel project and scope, Cochlear therefore started a paediatric registry (P-IROS) in 2015, a description of which is available in Sanderson [32].

### Selection of measures

Consistent with the aim of the registry, it is necessary to consider language and culture, that is, the measures must have the potential to be adapted cross-culturally. The IROS registry utilized two Patient Reported Outcome Measure (PROM) questionnaires to capture and represent real-world experiences. General demographics, hearing history and other patient-related characteristics were captured via clinical case report forms. This selection impacted IROS in two ways: firstly, the requirement for pre- and postoperative assessment due to the nature of the questionnaires that concerned the current perceived status of hearing and secondly, the absolute need for long-term repeated-measure outcome data, particularly for CI recipients, because optimal benefit from the hearing implant might only be obtained after several months. We concluded that the postoperative measures needed to be suitable for repeat assessment at several annual time points up to 3 years following implant.

### Type

All registries are in essence observational, collecting data from routine clinical practice and therapy, but they can be implemented as voluntary or mandated. Inherent in a corporate-sponsored registry like IROS is that it must be voluntary [18]. It can be a so-called patient registry, which systematically collects data concerning a defined treatment population, or a study registry that is designed to answer specific research questions or provide supporting evidence. IROS was designed and implemented as a patient registry focusing on real-world patient-related benefits.

### Stakeholders

Stakeholders share common interests but also have specific expectations [17]. Consideration should be given to the scientific community, healthcare professionals, health economists, healthcare payers and reimbursers, healthcare authorities, regulatory agencies, medical device manufacturers and, often not sufficiently considered, patients and their disease associations. Nevertheless, it is important that the registry design and implementation are not overloaded, to enable delivering on its primary objectives, which may require a compromise between stakeholder interests. Once defined, the success of the registry will be determined by clear communication that enables coordinated efforts and well-defined ongoing measures of success. We note that our stakeholder group grew as the utility of the registry became more apparent.

### Governance

The overall governance of a registry lies best within a small steering committee, including the project manager. The steering committee interacts with stakeholders and gives particular attention to documenting ethical approvals, data entry, and retrieval and monitoring procedures [24]. This is especially significant in a diverse and wide-reaching international corporation. A choice needs to be made between in-house governance and contracting an external contract research organization (CRO). The latter may facilitate rapid changes but, in our experience, may not always be practical from a cost or resource point of view.

### Degree of flexibility

Part of good planning is deciding how long the registry will run or defining when it will end in terms of the required number of data points meeting set objectives or the criteria for termination (such as poor recruitment or follow-up rates). Amendments are necessary for technical updates (browser version, etc.), data transfers and expanding the registry to meet stakeholder requests and to incorporate changed regulatory demands, which are to be expected. For instance, the IROS was adapted to provide mandated post-market clinical follow-up (PMCF) data for medical devices, initially, in 2014, under the EU Active Implantable Medical Devices Directive (AIMDD) [90/385/EEC] [11] and more recently under EU Medical Device Regulation [EU MDR 2017/745] [31]. Prior to the initial enrolments in 2011, the IROS registry was designed to meet the requirements, for the protection of patient personal information and to be registered with the Commission for Data Privacy in Belgium on behalf of all European countries (CPVP) [10]. Subsequently, further refinements were made to ensure compliance with the EU General Data Protection Regulation (EU GDPR 2016/679) [16] requirements in 2018. It is crucial to keep the registry up to date with the regulatory environment; however, there are no harmonized regulatory requirements for registries across countries [2].

Deciding on the operating term of the registry and keeping to a minimum duration required will help avoid the accumulation of regulatory imposed changes, which may lead to further cost and resource drain for registries whose duration is open-ended.

We should add that addressing evolving commercial needs, such as the release of new products and their iterative inclusion in an established registry platform, may equally impose resource-consuming changes to documentation, training and support. Before making such changes, it may be worth considering whether the evidence landscape has evolved to warrant a new registry design and concept.

### Transparency

Transparency means letting stakeholders know how and why data are collected and providing a means to access that data. Data ownership is an inherent part of transparency, privacy and security.

Transparency has become more relevant in study design over the last decade [2]. For the IROS, it was communicated and understood that clinics had full access to their own data for analysis and reporting as desired and that collaborative permission from other participating clinics was required to access and use any other group’s data. Furthermore, Cochlear owned all data for analysis and reported to stakeholders as required. Transparency also carries the responsibility of assuring data anonymity when data reporting to any parties.

### Legal and ethical aspects

It is important to determine whether patient consent needs to be obtained from each patient, or whether anonymization is sufficient [5, 38]. When designing a patient consent form, it is important to clearly indicate that the analysed data will be used for publication and reports, as well as any secondary uses of data (e.g. data mining or exploratory research). It is equally important to emphasize that any published data will maintain patient privacy through anonymized group reporting. It is beneficial to define the potential data uses broadly enough, already foreseeing potential data analysis requests outside the primary registry objectives. A clear definition of the purpose of end-use for the data collected is also a requirement by EU GDPR. If registries are designed to not collect patient identifiable information and the data collected is fully aligned with clinical routine, it might be possible to obtain a waiver from ethics committees, allowing data collection directly from the patients without going through the informed consent (IC) process. This tremendously reduces the burden of ongoing compliance on a project. Another possibility for streamlining registries is to use questionnaires that can be responded to directly by patients via marketing-type surveys, without the involvement of a clinician in the data collection. We recognize that certain detailed clinical data that requires the health care professional’s input will not be accessible via this route.

### Financial commitment

**Fig. 2:**
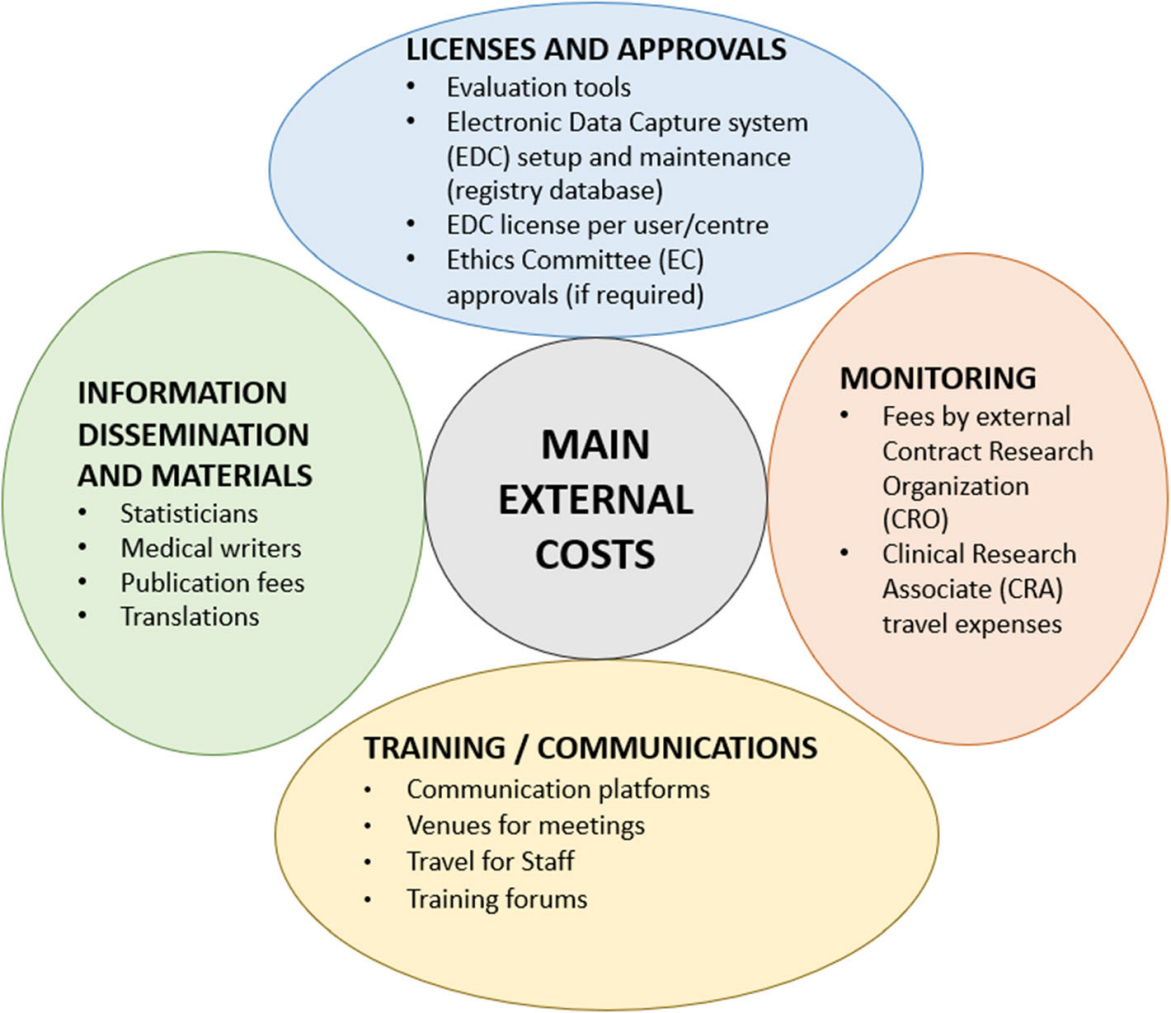
The main external cost items

As a long-term project, it is important to properly assure financial resources. Design, implementation and maintenance costs, including upgrades as needed, are concerned with different facets of the registry (Fig. 2). Even before starting the data collection, adequate investment plans are needed for all levels of documentation that may include translation of test tools, providing various information for ethics committees in local languages, information materials for clinics and patient participants and various other communications. Other activities such as the development of the database, webinars, travel costs and in-house registry support personnel must also be considered. The database, also called an Electronic Data Capture (EDC) system, may need to undergo technological iterations over the years, which is an aspect to consider and financially plan for.

### Costs

To illustrate the above, what follows is an overview of the key costs incurred over time by our registry specifically. The main costs were incurred in the development and implementation, including the initial set-up of the EDC platform for the first group of languages, with English as the main version. Subsequent costs included translation and adaptation into additional languages and providing a multicultural platform for data capture. This was followed by ongoing costs including annual platform maintenance fees, amendments and modifications to the existing forms to include newly approved products into the registry, access fees for new users at new and existing clinics, inclusion of new product-specific forms where applicable, EC approval costs for new sites and extensions of EC approvals where applicable.

In the initial stages, each new participating site was trained either via collective group meetings where possible or during one-on-one site trainings for clinical staff to be involved. The training was performed by responsible project managers and coordinators globally and regionally. As time progressed and the number of on-board sites grew, training was implemented remotely via live online sessions. This was considered a more sustainable and cost-effective option over time. Furthermore, after training, the clinics were provided with training materials either electronically, via the registry platform, or in hardcopy. This enabled refreshment of the training at their convenience and as new users were recruited at a participating site.

Following the original monitoring plan, primarily remote monitoring of the data collected in the EDC was performed. A sample of registered implant users was monitored to carry out the planned risk-based monitoring (i.e. approximately 5% of enrolled subjects). The aim of the monitoring was to confirm patient consent dates and assess the accuracy and completeness of the data entered. As certain products were included in the registry under active post-market clinical follow-up (PMCF) plans, remote monitoring was extended to all users of these devices. In these cases, on-site monitoring was performed if the need was indicated after remote monitoring. These were used as an opportunity to both monitor and retrain staff where needed. Hence, while costs were initially relatively low for monitoring tasks planned at registry launch, as the number of products under PMCF plans increased, costs for travel and staff time steadily increased, subsequently incurring further costs through enlisting monitoring services via an external CRO.

## Implementation

### Recruitment

Participating clinics may be recruited directly by the company, its local distributors or via interest groups. Patients are typically recruited by the clinical professionals involved in their care and treatment; thus, their selection criteria can be carefully delineated. For the data to be representative, aim to enrol enough patients from different clinics in different countries. However, this should be balanced against the costs of translation and of implementation in different regulatory regions.

### Ethics approvals

All sites were asked to obtain ethics committee (EC) approval prior to recruitment of the first subject. A national EC approval, covering one or more sites in the same country, was obtained in five participating countries: Colombia, France, Hungary, Spain and Turkey. A national EC waiver was provided upon application for Poland and the Netherlands. Other countries required local EC applications at each site, resulting in 27 local EC approvals. These included Argentina, Austria, Belgium, Brazil, Germany, Portugal, Slovenia and South Africa. An EC waiver was also provided by some local clinics in Germany upon application. No applications for EC approval were rejected. Not all clinics with EC approval proceeded to enrol at least one implant recipient in the registry. The reasons for non-activity in the EC-approved sites were not explored. In some countries and select clinics (e.g. Argentina and Germany), ECs required a specific consent for minors (i.e. legal guardian’s signature). EC approvals at most sites were time-limited and needed renewals in case of expiration.

### Data entry

Clinics should be vetted for appropriate infrastructure for data input and resources to provide long-term follow-up. Data entry is quite a burden when added to everyday clinical tasks. Data entry might be a standard expectation for a clinical trial but not so for a voluntary registry. During the clinic selection process, it is therefore necessary to clearly define and confirm reciprocal responsibilities and expectations. We chose, at the outset, to notify clinics that they could review the proposal to participate online, and subsequently register and receive access to the registry platform. Registries are suitable projects for investigators with various levels of experience. Indeed, relatively inexperienced investigators can participate as researchers in registries. Such investigators may, however, lack proficiency in regulatory aspects and may need more assistance to maintain compliance with documentation and protocols.

### Incentives

To motivate ongoing participation, all contributing stakeholders will need general incentives. The registry can be offered as a service that provides a means for the clinics to collect data systematically and access their own collated data for publication or patient counselling, as well as be part of the collective research on a larger scale.

### Clear documentation

*Clear documentation* means developing concise and easy-to-understand materials that assist in fulfilling all aspects of a registry. It involves the creation of the master protocol, registry agreements, master versions of all instructions and report forms, data-entry portal frameworks, etc., all of which may require translations for international registries. There must be checks and validation of the accuracy of translations and any appropriate cultural adaptations. This can be time-consuming and costly. In some regulatory domains, the registry may be considered a retrospective study, in others as non-invasive, but still a clinical study. This can have implications for additional documentation requirements (for example, applying the ISO 14155 standard to a registry ‘study’ under EU MDR 2017/745).

### Data collection procedures

It is important to make every effort to reduce the barriers to data entry. The process through which data is collected should be easy, seamless and require as little time as possible and, to be sustained, the data requested needs to be concise. Including a paper-and-pen mode of collecting data is potentially more time-consuming, requiring report forms to be printed, given to participants, and then re-entered, often by the clinic personal, into the registry’s web portal. Re-entry of data may not only generate entry errors but is time-consuming and may lead to reduced compliance over time, especially as more subjects are enrolled in the registry [38].

## Ongoing management

### Data management

If it is deemed necessary to have external builders/contractors for the database, it is essential to keep an overview of data completeness and quality through diligent monitoring and reports. Dashboard views available to the steering committee aid in spotting problems as early as possible so that timely rectifications can be implemented. It may be possible to build-in recruitment targets and use risk-based triggers, for initiating monitoring actions, to improve recruitment or increase data completeness. The registry platform should establish a fully automated electronic audit trail to allow traceability of all changes to data entry made over time, including by whom.

### Data completion reporting

We learned that the best option was a combination of automated and more frequent reports of data completion for registered patients. Also, we kept informing clinics that all data intervals were acceptable; that is, skipping a test period does not eliminate that subject from later analysis, so long as baseline data had been collected. Strict oversight of missing data and attrition rates is paramount for the registry to quickly identify issues, implement mitigation and ensure delivery of its objectives.

### Monitoring

A monitoring plan needs to be in place before data collection begins. It includes monitoring on a site-by-site basis at, either all or select, participating sites. Monitoring involves oversight of all administrative and regulatory aspects to do with participation in the registry. Keep in mind that applying compliance requirements, normally associated with clinical investigations, may overload a voluntary registry and cause failure to deliver. Monitoring, which can be done on site, remotely or a combination of both, should occur at regular intervals throughout the life of the registry [17]. Also, the budget impact of monitoring activities should not be underestimated, as already indicated in the ‘Cost’ section above.

### Training

Upfront planning of an appropriate mode of training that can readily reach all contributors is key. Often, this may require the availability of online training platforms and specific guidelines in different languages, as contributors will be widely distributed nationally and internationally. Local visits by sponsor representatives, as well as question-and-answer regional webinars will help to confirm the efficiency of data entry and follow-up. Trainers should introduce the relevance of the registry along with the registry protocol and its procedures.

Instructors should also describe the outcomes and potential opportunities for reports and publications. They should also emphasize why complete datasets are more powerful and remain essential to the goal of the registry [38]. Regular newsletters, summaries of reports and publications, yearly investigator meetings and refresher training sessions can add to a sense of shared responsibility in the study group, and help increase compliance to data provision and procedures. Training records should be collected to maintain oversight.

### Personnel on and off-boarding

Registries running over a long time frame will see staff changes at participating sites as well as in the sponsoring organization. Clear processes for the contributor on- and off-boarding are required, including creation and closure of accounts, on-boarding training and respective documentation. A part of the off-boarding process must be a critical check of all necessary documentation filed (i.e. CVs, training logs) as retrospective requests may not be possible.

### Financial commitment

Registries may suffer from insufficient funding, especially if they run over a long period of time. Sponsoring a registry requires long-term vision to see the project through delivery on its objectives and acknowledgement of the high costs involved [4]. It is, therefore, important to have regular progress reviews with the sponsors, ensuring the registry is delivering on their expectations [17].

### External and internal audits

Audits are concerned with checking the overall study conduct at the sponsor level, ensuring adequate study oversight and documentation, according to the sponsor’s standard operating procedures (SOP), which in turn should now be compliant with ISO 14155:2020. Audits are therefore associated with quality assurance [23, 30]. These audits ultimately required much more oversight than anticipated for the IROS. Internal audits are voluntary, initiated by the sponsor and eventually conducted by a qualified consultant. Audits are valuable in informing the sponsor of potential compliance weaknesses at any given time, allowing for rectification in a timely manner. This can prevent more drastic actions as a result of an unannounced (external) audit, for example, by a notified body.

### Readjusting registry objectives

Any registry will have been set up to meet certain objectives. Over the course of the registry, these objectives may shift and require readjustment. In our case, the initial objective of the IROS was to survey the hearing performance of implant users. A few years into the course of the registry, regulatory mandates for implantable medical devices [AIMDD 90/385/EEC] required active collection of PMCF data for certain implant devices. In the ensuing assessment, the IROS was deemed a suitable tool and source for such data collection. New privacy regulations EU GDPR in 2018 however imposed more scrutiny on how clinical data could be collected and stored, increasing the burden of conducting the registry, by for example, the need for a revised informed consent process. Later, in 2020, the EU MDR [2017/745] newly required that PMCF data should be collected on a broader scale than suitable for IROS.

At the same time, the registry concept is now better represented in ISO 14155:2020 as a post-market, observational and non-interventional study type. This should lessen the burden in certain key areas for conducting such a registry compared to requirements in ISO 14155:2011, which did not cater well for registry activities, nor the reporting of registry data to regulatory authorities. Thus, registry objectives may change in the course of a registry and change the course of the registry or ultimately even determine its closure. Such changed objectives can be imposed externally, e.g. due to regulatory mandates.

## Data analysis

### Statistics

Statistical analysis should be planned and described in the planning phase, keeping in mind that a long-term registry acquires substantial amounts of data. This abundance potentially allows stratification by patient profile. Registries offer an opportunity to mine the data for the analysis of different subgroups with greater statistical power. It is, however, essential to first address the primary goals of the registry, while being open to learn from interesting and valuable new information that may be revealed. Non-inferential data ‘analytics’ can be useful, but high-quality statistical analyses should be the main aim. We note that this can be time-consuming and expensive.

### Measures of success

These are concerned with being able to make projections as to how the data will unfold and further guide the planning of monitoring activities. As progress is checked against specifications of the protocol (goals), it is important to interact with each centre to address deficiencies and data problems and to then make adjustments promptly. For Cochlear, the measure of success was primarily the capture and report of sufficient patient-related longitudinal outcome data from a broad population of implant recipients, to represent benefits in the real world. This enabled further analysis and interpretation of treatment benefits for the various patient subgroups involved.

### Missing data

It is not possible to mandate data entry for a commercial patient registry; therefore, it is wise to anticipate and account for a greater attrition rate than in rigorous clinical trials [18]. This can be factored in when considering the endpoints of the registry. There should be rigorous statistical analysis of data sets of enrolled individuals that are followed up versus those that are lost to follow-up [21, 22]. It is also prudent to consider accounting for aspects that may influence or skew the interpretation of the data in a voluntary registry. The report should consider how a registrant was recruited, the guidance provided to subjects on how forms and questionnaires should be completed, by whom, and the timing of the evaluations.

The IROS registry had a mandated follow-up period of 2 years post-implant with an optional 3rd year follow-up. Subjects were recruited throughout the lifetime of the registry. Long-term follow-up, after implantation, was desired to compare to hearing and quality of life status at baseline before implant. It is known that the greatest performance improvements in hearing function for adults and adolescents with post-linguistic deafness are anticipated in the first year after implant [21, 22]. Longer term follow-up to assess ongoing stability of post-treatment benefits, while of interest, was considered outweighed by the added burden upon participating clinic efforts. Experience has shown that follow-up of enrolled implant users significantly diminished from 1 to 2 years and further between 2 and 3 years post-implant [21, 37].

While not the subject of this paper, a separate paediatric registry (P-IROS) was launched after the more general IROS. This registry focused on the recruitment of deaf-born or deafened children between the ages of four and twelve. As speech and language skills evolve, post-implant, during early years through to school age [15], the P-IROS aimed to collect self-assessed outcomes via parent and clinician proxy for up to 5 years after implant for each registered child. A similar diminishing return was reported for data capture after enrolment and then also after the first follow-up year [34].

### Data access

In addition to the statistical analysis strategy driven by the registry sponsors, contributors need to have access to their own data in a comprehensive form and format [17]. This may include raw data access in a compatible form that can be used with common statistical packages. However, our experience revealed that investigators greatly appreciated being able to request data reports in Word, PowerPoint, etc., which they could utilize in their own locally generated reports and presentations.

## Closing the registry

### Challenges

Over the course of the registry, we observed a steady decline of motivation and participation by the investigators. Out of 77 initially recruiting clinics, only 10 were active in the final year of the registry. Follow-up data entry beyond year 1 had also steadily declined. We therefore gathered feedback from the remaining active investigators on their motivation for continuing. It was found that investigators struggled with access issues to the EDC due to increasing incompatibility of the EDC platform with current technology (i.e. up-to-date browser versions). Clinicians were also challenged by their heavy workloads, preventing them from continued participation. Many had collected paper versions of the patient questionnaires and were not able to find the time to enter the resulting data into the EDC. Study nurses needed to be recruited for data entry. A few clinicians were only interested in certain data points of the questionnaires and not the complete set, lowering their motivation. Furthermore, clinicians changed roles, and their successors did not share the same interest in the registry project. It also became increasingly challenging to continue maintaining the participating site’s ethics approvals, their reporting, expiry and renewal timelines, and the collaboration of the investigators to fulfil these obligations.

### Closure timing

A registry should have a defined lifespan, with trigger points for the reappraisal of the needs and conditions for continuation. In the case of the IROS, we chose to close the registry for several reasons, including the challenges outlined above, additional changes in regulations and related documentation requirements; a decline in patient recruitment and follow-up; and changing requirements for PMCF.

### Database closure

Clinics need to be informed of the decided upon pending closure of the database without delay. This is somewhat the opposite of the case for the planned closure of clinical studies where all subjects are required to complete evaluations according to the clinical protocol. Initially, the database should be frozen to allow for data queries to be answered before the database is closed.

### End-of-study report

It is good practice to produce a final report which describes and analyses all the data collected according to the original registry plan. In some regulatory environments, the registry may have been granted approval as a clinical study and, thus, the required clinical investigation report (CIR) will need to be filed within a prescribed time frame (e.g. 1 year) after closure. Notifications and reports to approving ethical committees will be required at study closure.

### Publication

Some aspects of the registry data may be of more interest than others for publication. However, as for clinical studies, it is advisable to publish as much as possible of the primary and secondary outcomes from the registry for all or select patient groups, both for transparency and to share the learning and knowledge gained. In addition, summary reports describing the registry findings may need to be completed, where the registries have been listed on public clinical trial portals.

## Outcomes

As well as providing considerable learning about the conduct of a company-sponsored registry, the IROS registry was considered a success in terms of having achieved its goal in providing a view of real-world benefits from hearing implant treatment.

The IROS registry succeeded in enrolling a large cohort of users of an implantable hearing solution, in particular CIs, who provided longitudinal real-world data. These data were utilized at various intervals to generate a wide range of local and international conference posters or presentations as well as white paper articles and publications in peer-reviewed journals. Scientific findings describing outcomes for CI recipients as captured via the IROS registry resulted in five publications [12, 21, 22, 28, 37]. A summary of the results from the IROS registry following its closure is also publicly registered [ClinicalTrials.gov NCT02004353]. Pooled data was used to identify trends in outcomes as well as provide input for the design for more robust comparative studies.

Furthermore, IROS serves as an example of a large prospective observational study design yielding meta-analysis possibilities. The available data from adult CI users was used to investigate the potential to reduce the number of SSQ49 questions administered, while maintaining the required sensitivity to changes in self-reported hearing benefits over time. Statistical comparison was made between outcomes measured using the complete long-form SSQ49 and those measured by extracting the subset of 12 questions in the SSQ12 [29]. The analysis confirmed clinical equivalence between the long- and short-form questionnaires and thus a potential to save response time [37]. Consequently, Cochlear now uses the SSQ12 for studies in the field of implantable hearing solutions.

## Recommendations for the future

There is an abundance of recommendations for developing a registry [17] available, but given our 10-year experience, we can succinctly focus on four points for distinct recommendations.

### Reduce site workload as much as possible

Moving forward, two options are either to establish a clinic-based registry, where source data derives from information collected by investigators, or alternatively to utilise directly generated patient-sourced data. The first approach encounters the greatest challenge we experienced, namely insufficient resources in some sites. Since the IROS deployed a combination of clinician-reported and patient-reported data collection (via PROM questionnaires), our recommendation is to focus on employing questionnaires that can be directly filled in by patients [24], without the need for a clinician to be involved with data entry.

### Develop an interactive registry

Utilise web-based data entry with a simple interface, the operative word being ‘simple’. The more automatization in data collection and management the better; nonetheless, this will come at increased design and implementation costs. An online chat advisor to participants and clinics to support data entry may be useful, as well as something such as a hotline for those having special enquiries. Choosing a Software as a Service (SAAS) registry platform provider might be of advantage, as a state-of-the-art provider will ensure their platform stays current with new developments, including evolving regulations for data privacy. A truly interactive registry, however, is better supported by newer technologies only available within the last decade [9].

### Utilise modern-day digital communication modes

As early as 2016, social media platforms became a potential medium for data collection [36]. Social media is widely utilized by individuals of all ages, via mobile devices that include smartphones and tablets [8, 27]. As such, these devices present an opportunity, for further consideration and leverage, as data entry tools for health-related data, within today’s communications environment. In a study conducted on this topic, it was concluded that it is an efficient means for patients to enter their own data, particularly QoL data, and that participants are keen to contribute to data entry via apps [24].

Cochlear has already successfully instituted several proprietary applications for use as patient surveys. Issues in addressing patients directly must be considered and overcome, by various methods. For example, making information, obtained from patients, directly available simultaneously to their health care professional, and as appropriate, to the sponsoring company. At the simplest level, there only needs to be appropriate access control and automated announcements and reminders for survey completion. Developing an app for remote responses may lead to the creation of new and better registries using modern data retrieval methods. The future of international registries lies in direct digital interaction with patients and the reduction or elimination of clinic overload. The idea is to provide ease of data entry at any time and place; to be able to partially enter data and complete at the patient’s convenience. This may lighten the load of data entry for the patient by offering more flexibility and choice as to how and when data is provided, ultimately increasing patient empowerment. Note that the ability to use an app could represent a built-in bias: participants must have the financial means to own a smartphone and be able to use the technology.

### Find the most efficacious means to motivate all participants with judicious feedback

The registry sponsor should consider and monitor the motivation of clinics and what their rewards might be for committing to the patient recruitment and data collection process. Rewards include publication and access to data. Affirm progress towards agreed milestones that may include patient enrolment, complete data sets, commitment to publish papers or present data over a given period of time and offer assistance in their creation. This active implication may serve as an incentive to investigators to continue to collaborate. Equally, patients may see benefits by self-monitoring their progress over time on self-reported measures, having appropriate access to their own data in an understandable summary format. More frequent and relatable feedback may also help incentivize patient responses over time which may be possible via smartphone apps and or home-based computers.

Regardless of whether it is a traditional registry or one run via a specially designed application for mobile devices, it is worthwhile to run a pilot test of implementation.

## Closing remarks

We have shared and described our experiences and learnings based on the design and implementation of a multinational, multicultural, registry from a single manufacturer, with the aim to help others, embarking on such a registry, consider all aspects involved. It is no small endeavour to collect a large dataset over an extended observation time frame that is representative of a treatment in a large population pool, especially one that is voluntary. IROS developed, maintained and effectively captured and harvested data through an international registry. It was the first international registry in the hearing field. Pioneered by Cochlear, it provided evidence from longitudinal self-reported, patient-related outcomes. The design and initial small-scale implementation proved to be relatively easy compared to its longer-term implementation on a much larger scale. In particular, maintenance of required updates, cultural adaptations, sustaining data entry efforts for both new and existing registrants, performing data analysis, and providing reports all demanded significant resources. We propose that it is necessary to focus resources to best support the primary end goals. Selecting and adapting meaningful self-reported patient-related outcome measures, coupled with data capture from large and small clinical facilities across countries and languages has enabled meta-analysis of the collective longitudinal data. The information provided has led to a better understanding of real-world benefits including hearing in daily life and generic quality-of-life benefits after treatment with hearing implant solutions, primarily CIs for a broad treatment population.

While this report reflects our registry experience, support and costs in absolute terms for any registry will depend on many factors. This will include the registry design, the data capture platform chosen, requisites on EC applications and renewals, monitoring requirements, the number of sites and regions involved and the costs structure in these respective jurisdictions and of course the intended duration of the trial.

## Data Availability

Not applicable
